# IEEE 802.11ax OFDMA Resource Allocation with Frequency-Selective Fading

**DOI:** 10.3390/s21186099

**Published:** 2021-09-11

**Authors:** Sergei Tutelian, Dmitry Bankov, Dmitri Shmelkin, Evgeny Khorov

**Affiliations:** 1 Institute for Information Transmission Problems, Russian Academy of Sciences, 127051 Moscow, Russia; tutelian@wireless.iitp.ru (S.T.); bankov@iitp.ru (D.B.); 2 Mathematical Modeling Lab, Huawei Moscow Research Center, 121614 Moscow, Russia; Shmelkin.Dmitri@huawei.com

**Keywords:** Wi-Fi, 802.11ax, scheduling, frequency-selective fading, power allocation, CSI estimation

## Abstract

This paper studies the usage of orthogonal frequency division multiple access (OFDMA) for uplink transmissions in IEEE 802.11ax networks. OFDMA enables simultaneous multi-user transmissions in Wi-Fi, but its usage requires efficient resource allocation algorithms. These algorithms should be able to adapt to the changing channel conditions, including the frequency-selective fading. This paper presents an OFDMA resource allocation algorithm for channels with frequency-selective fading and proposes an approach to adapt the user transmission power and modulation and coding schemes to the varying channel conditions, which is efficient even in the case when the access point has outdated channel state information. The proposed scheduling algorithm and power allocation approach can double the goodput and halve the data transmission time in Wi-Fi networks even in dense deployments of access points.

## 1. Introduction

Wi-Fi networks have become an integral part of modern life. They are used in many scenarios; the number of hotspots is continuously growing [1], which increases the density of the network and the interference. Recently, the IEEE 802 LAN/MAN standardization committee issued the 802.11ax [2,3] amendment to the Wi-Fi standard. In contrast to the previous 802.11ac, 802.11n amendments that mostly featured the increase in the nominal data rates, 802.11ax focuses on improving the efficiency at the user layer.

The 802.11ax standard [4] describes many innovative solutions designed to improve the user layer efficiency, such as faster modulation and coding schemes (MCSs), overlapping basic service set management, spatial reuse, new power management solutions, uplink multi-user multiple-input multiple-output (MIMO), and orthogonal frequency division multiple access (OFDMA), which is the focus of this paper. OFDMA allows dividing the available channel by frequency between different users and thus enables several stations (STAs) to transmit or receive data simultaneously. OFDMA enables more flexible usage of channel resources in comparison with the legacy non-OFDMA transmissions. Moreover, for uplink transmissions, OFDMA allows concentrating the STAs’ transmission power in narrow frequency sub-bands and, consequently, using faster MCSs.

Naturally, the benefits of OFDMA for Wi-Fi depend on the method of splitting the channel resources between the STAs, i.e., on the channel resources scheduler. One of the simplest ways—the legacy approach—is to allocate the entire channel to only one STA at a time. Such an approach might be inefficient when many STAs need to transfer small amounts of data because much channel time is wasted by the transmission overhead, so the schedulers that exploit the possibility of simultaneous transmissions by several STAs should be considered. This paper focuses on uplink OFDMA, but most of the obtained results can be generalized for the simpler downlink case.

OFDMA schedulers are well studied in the literature for scenarios of a general wireless communication system [5,6], where the scheduler is designed as a solution of an optimization problem, e.g., to allocate tones and transmission power to the users in order to maximize the total rate while keeping the total power below some threshold. However, most of such research considers idealistic scenarios when the transmitter can be assigned to an arbitrary set of tones, the transmitter and the receiver know the channel state information (CSI) perfectly, and the transmitter power can be arbitrarily distributed among the tones. Such assumptions and simplifications allow us to find exact solutions to optimization problems, and the computation time required to solve the scheduling problem depends linearly on the number of users, tones, and MCSs [6], but real communication systems impose additional restrictions which make the problems more complex and require new solutions. For example, in LTE, the whole channel is split into several resource blocks of the same size. An LTE base station can allocate an arbitrary set of resource blocks to a user equipment for downlink transmission, but for the uplink transmission, the set of resource blocks shall be contiguous because of the usage of Single-Carrier Frequency-Division Multiple Access (SC-FDMA). The uplink scheduling problem becomes NP-hard because of the requirement [7], so the existing research focuses on designing some heuristic algorithms to maximize selected utility functions. At first sight, these schedulers can be easily applied to 802.11ax, but in reality, in 802.11ax, the restrictions are more complicated: we cannot allocate an arbitrary range of 26-tone resource units (RUs) as it is done in 3GPP LTE. Thus, the schedulers designed for cellular OFDMA systems cannot be directly adapted to additional restrictions of 802.11ax, and we need to solve the scheduling problem taking into account the peculiarities of 802.11ax uplink OFDMA.

Various papers about 802.11ax divide the scheduling problem into several steps [8]: splitting of the frequency band into resource units (RUs), assignment of these RUs to the STAs, and selection of the MCSs that the STAs shall use for data transmission in the allocated RUs. While performing these steps, an AP should consider the amount of traffic that the STAs can transmit, the channel quality between the AP and the STAs, and the utility function that determines service priority. Moreover, the AP should abide by the standard limitations related to the positions and sizes of RUs [4] and with the requirement that the power of signals from transmitting STAs should be equalized with an OFDMA transmission [9].

An important feature of the wireless channels that complicates the scheduling problem is the frequency-selective fading. With frequency selectivity, various parts of the frequency band experience different attenuation. Although frequency selectivity is neglected in many papers that study the schedulers for 802.11ax, the lessons learnt from LTE optimization confirm that it is essential to design an OFDMA scheduler that can deal with it, assigning RUs with the most significant channel gain to each STA.

Another problem often disregarded by the existing research is that the AP should know CSI for every STA and every sub-band to make proper resource allocation. However, perfect channel knowledge is not available in the real world, and a proper resource scheduler for OFDMA in Wi-Fi should consider that the AP might have outdated CSI. At the same time, most research related to OFDMA scheduling for 802.11ax assumes that the AP has ideal CSI knowledge.

The contribution of this paper is threefold. First, we develop a resource scheduler for OFDMA in 802.11ax networks, which is efficient for frequency-selective channels. Furthermore, the scheduler considers the implementation-conditioned restriction that the power of the signals from the transmitting STAs should be the same at the AP. Second, unlike existing research on OFDMA scheduling for 802.11ax, we consider the case when the CSI is updated at the AP with some period, investigate the impact of outdated CSI on network performance, and propose an algorithm for tuning available CSI estimates to match real channel conditions. Third, we evaluate the performance of the proposed solutions in a dense network scenario with multiple interfering APs.

The rest of the paper is organized as follows. In Section 2, we observe the OFDMA basics in the 802.11ax. Section 3 reviews the works related to the OFDMA scheduling in Wi-Fi. Section 4 contains a statement of the scheduling problem. In Section 5, we propose a greedy approach to solve the noted problem. In Section 6, we examine the power allocation issue and describe a CSI adaptation algorithm. Section 7 presents and discusses numerical results. Section 8 concludes the paper.

## 2. OFDMA Transmission

**Figure 1:**
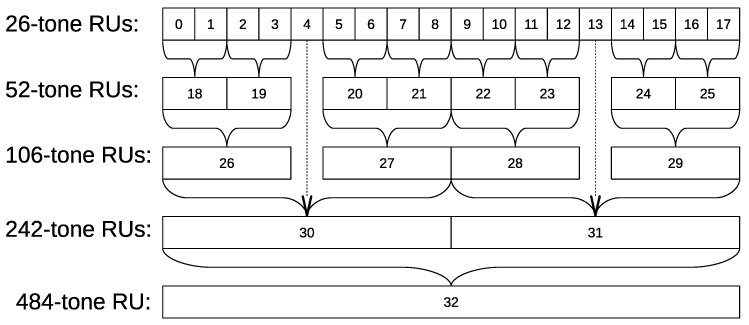
RU structure in 40 MHz channel.

In 802.11ax, the whole channel is split into sets of subcarriers (tones) called resource units (RU). An RU may consist of the following numbers of tones: 26, 52, 106, 242, 484, 996, and 2 × 996. The available sets of RUs depend on the channel bandwidth. For example, the RU structure for the 40 MHz channel is shown in Figure 1. A 484-tone RU occupies the entire channel and consists of two 242-tone RUs. A 242-tone RUs consists of smaller RUs and so on. Such an RU structure makes OFDMA scheduling in 802.11ax networks different from scheduling in LTE, where a base station can use any set of resource blocks for downlink transmissions to a user, and any contiguous set of resource blocks for uplink transmissions by a user.

According to the 802.11ax standard, both in the downlink and uplink, OFDMA transmissions occur on a per-frame basis. It means that different frequency parts of a frame are sent to or by various STAs, in the case of a downlink or uplink transmission, respectively. In both cases, the AP determines the duration of the OFDMA frame and allocates RUs to the STAs that will transmit or receive data in this frame. Each STA may be assigned to no more than one RU that may have any available size (26-tone, 52-tone, etc.). The AP also sets the modulation and coding scheme (MCS) that the STA shall use. The amount of transferred data depends on the RU size, frame duration, and MCS. The wider RU and the faster MCS, the more data can be transmitted per time unit. Table 1 shows the data rates for different RU sizes and MCSs. Note that high-speed 1024-QAM MCSs are available only in the wide RUs.

To point out the implications of the 802.11ax RU assignment and its difference from LTE, let us consider a simple scenario when an AP has to schedule resources to two STAs in a 20 MHz channel. The RU structure in 20 MHz channel corresponds to one 242-tone RU in Figure 1. The AP can allocate the entire channel to one STA or divide the channel between two STAs. However, the 242-tone RU cannot be divided into two arbitrary parts. Unlike LTE, 802.11ax allows either to assign the whole band to one STA or to assign a 106-tone RU (or a smaller one) to each of the STAs. In the latter case, whatever RUs are chosen, at least a 26-tone RU is wasted. As a result, instead of selecting the best RUs for each STA one by one in a greedy way, as it is typically done in cellular systems, the AP has to search for allowed RU configurations and the ways to assign given RUs to the STAs.

In 802.11ax, the transmissions within one OFDMA frame shall start and end synchronously. While it can be implemented relatively straightforward in the downlink, uplink OFDMA transmissions require additional control from the AP. As this paper focuses on uplink OFDMA transmissions, let us consider them in detail.

Uplink OFDMA transmissions are fully controlled by the AP. To start an uplink OFDMA transmission, the AP sends a trigger frame (TF). It specifies the resource allocation for the STAs: the MCS, power level, RU, frame duration, and other parameters. Short Inter-Frame Spaces (SIFSs) after the STAs send their data in the allocated RUs. Finally, the AP replies with an acknowledgment frame called Multi-STA BlockACK.

**Figure 2:**
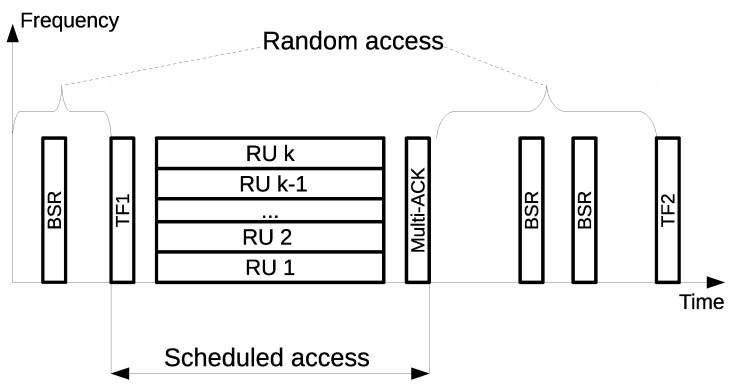
An example of uplink OFDMA transmission in 802.11ax.

A general view of the uplink transmission process is shown in Figure 2.

To schedule resources, the AP needs to know which STAs have data for transmission. For that, the STAs report to the AP that they want to transmit data using the Buffer Status Report (BSR) frames. After receiving the information about the STAs’ buffers status, the AP allocates resources and sends the TF.

The optimal RU and MCS allocation depends on the signal-to-noise ratio (SNR) at the receiver. Fast MCSs and wide RUs require high SNR for successful transmission. Higher SNR can be achieved by increasing the transmission power, but the maximum transmission power limitation restricts the choice of RU and MCS.

Another issue that restricts the choice of RU and MCS is the fact that channels between the AP and STAs have different power gain, which means, along with the frequency selectivity, that the signals arrive with different power. If the signals have significantly different power spectral densities at the AP, the weak signals are received with errors [9]. To avoid this problem, the STAs should adjust their transmission power in such a way that the power level of the signals received at the AP is approximately the same for all simultaneously transmitting STAs.

## 3. Related Work

OFDMA is widely applied in cable access networks and wireless communication systems and has been studied for a long time. Many different schedulers have been designed for OFDMA: centralized and distributed, optimal and suboptimal, instantaneous and ergodic, cooperative and non-cooperative, and single cell and multicell [6]. In this paper, we focus on centralized single-cell scheduling. A typical scheduler for a general OFDMA system solves an optimization problem [5] to maximize some utility function, e.g., the total rate, by assigning tones to the users and setting the transmission power of the users on these tones. For some utility functions, the scheduling problem can be solved in an optimal way in a time that depends linearly on the number of users, tones, and MCSs [6]. However, the optimization problem for the scheduler becomes more complicated for uplink OFDMA transmissions and for specific wireless communication technologies. For example, in cellular technologies such as LTE, a user cannot be assigned to an arbitrary set of tones for transmission: because of the SC-FDMA scheme, the users can transmit only in contiguous sets for tones. In [7], it is proved that such an additional restriction makes the scheduling problem NP-hard, so the classic solutions to the OFDMA scheduling problem described in [6] become inapplicable. Instead, heuristic solutions are proposed for OFDMA uplink scheduling, e.g., to consider users one by one and to assign them resource blocks with the highest utility function in a greedy way if it fulfills the contiguity constraint. As we can see, the scheduling problem with more restrictions becomes more complex, and in case of 802.11ax uplink OFDMA new constraints are added: the sets of tones assigned to users can have only specific upper and lower bounds. As a result, we cannot add resource blocks to a user’s schedule one by one as it is done in the LTE case, so new solutions should be designed specifically for 802.11ax.

The 802.11ax standard introduces uplink OFDMA for 802.11 systems that enables simultaneous transmissions by multiple STAs and motivates much research on the resource allocation and scheduling for these mechanisms. In 802.11ax, uplink OFDMA can be used for deterministic transmissions—when the AP schedules RUs for transmissions by specific STAs—and for random access using the Uplink OFDMA Random Access (UORA) which implements a slotted Aloha-like access scheme with binary exponential backoff. Much research [10,11,12,13,14,15,16,17] on uplink OFDMA in 802.11ax is dedicated to the analysis and optimization of UORA while leaving the scheduled part of OFDMA transmissions out of scope or considering a very simplified model of it, e.g., assuming elastic traffic or that the channel is divided into equal RUs. Although random access is useful in some scenarios, e.g., when the traffic is rare or when the transmissions should be made with very low delay [18,19], still the usage of UORA inevitably leads to collisions and losses of throughput. This paper focuses on the scheduled OFDMA transmissions, which might provide more efficient channel usage.

Various schedulers for 802.11ax have been designed and investigated under different channel and traffic conditions to use the 802.11ax capabilities fully. In [20], multi-user uplink transmissions are considered. It is shown that 802.11ax outperforms 802.11ac in terms of throughput and latency. However, it is assumed that the channel conditions for all STAs are the same, and the scheduler simply divides the entire channel equally for each STA.

In [21], the RU allocation problem for users of different service categories (VoIP, video) is investigated. The authors consider such factors as network load, the quality of service parameters, and fairness indicators. They also propose an approach to predict the values of some of these factors using known regression methods (for example, *k*-nearest neighbors) if these values are rarely updated.

In [22], the authors investigate the performance achievable in 802.11ax WLANs, including the usage of MU-MIMO and OFDMA transmissions. The authors develop an analytical model to find the saturated throughput and consider such aspects as the sounding procedure and the aggregation. The authors demonstrate that single-user transmissions are less efficient than multiuser ones. At the same time, the aspects of resource scheduling for OFDMA transmissions considering the channel state variability at different STAs are not taken into account and left for further research.

In our previous work [8], we have studied several OFDMA schedulers which split the entire channel between several STAs. We have shown that using the ability of multiple STAs to transfer simultaneously reduces the flow transfer latency and significantly increases the throughput compared to the case when only one STA transmits at a time. However, this study is conducted without considering the channel frequency selectivity, and the schedulers from in [8] cannot utilize the frequency-selective fading to improve the spectral efficiency. In contrast, in the present work, we eliminate this limitation.

In [23], the authors design a resource scheduling solution for OFDMA transmissions in 802.11ax. The authors focus on algorithms that can efficiently split the RUs and allocate them to STAs to minimize a cost function. As a cost function, the authors consider either the transmission duration or the maximal padding length. Note that as in [8], the designed algorithms do not take into account the frequency selectivity of the channel and do not consider the power allocation problem.

In [24], a resource scheduler for 802.11ax uplink OFDMA transmissions is designed. The authors develop a closed-loop controller that considers the amount of buffered data for different STAs and the QoS priority of different traffic flows to determine the weighting coefficients of the proportional-based scheduler. In this solution, the authors also leave out of consideration the issues of transmission power management at the STAs and do not consider frequency-selective fading: all the RUs with the same size are equal, which limits the applicability of this solution in realistic scenarios.

In [25], the authors develop a scheduler that solves an optimization problem to maximize the throughput for both uplink and downlink 802.11ax OFDMA transmissions. To avoid starvation of some STAs that have unfavorable channel conditions, the authors introduce an aging mechanism. The authors evaluate the performance of their solution using the ns-3 [26] simulator in scenarios where UDP and TCP traffic is transmitted and show a significant improvement in throughput and average delay compared with the non-OFDMA transmissions. However, also the proposed solution does not consider the frequency selectivity of the channel and does not control the STA transmission power.

In [27], the author proposes a mechanism to estimate the channel for downlink MU-MIMO using the uplink OFDMA transmissions. The usage of MU-MIMO requires knowledge of CSI in an entire channel, so it is proposed to add a number of slots in the end of uplink OFDMA frame during which specific STAs will transmit channel estimation signals, one at a time. This scheme is proposed together with a scheduler of resources for data transmission in uplink OFDMA, channel estimation with OFDMA, and user selection in downlink MU-MIMO. However, the optimization problem considered in the paper does not take into account the fact that the RUs should not overlap: in case when the scheduler allocates two overlapping RUs, the author proposes to rearrange the RUs in a non-overlapping way, but does not specify how to do it. Furthermore, note that the proposed scheme does not comply with the standard.

In [28], the authors study the performance of OFDMA in 802.11ax networks with extensive simulation in ns-3. The authors consider scenarios of downlink and uplink transmissions in saturated and non-saturated conditions and determine the gain from OFDMA in throughput and latency, respectively. In this work, only static OFDMA configurations are considered, but still the obtained results show that with OFDMA, the median latency can be decreased from 5 ms to less than 1 ms. The authors also remark that uplink OFDMA has potential for significant performance improvement. However, in this study, the authors consider an idealistic scenario when all STAs can transmit with the fastest MCS and also do not consider frequency selectivity of the channel.

In [29], the authors develop a recursive channel splitting and resource scheduling algorithm for 802.11ax downlink OFDMA transmissions. The developed algorithm implements the divide-and-conquer approach, descending from wide RUs to the smaller ones and splitting the resource allocation problem into sub-problems for smaller RUs. It provides a suboptimal solution because when the problem is divided between RUs, the sets of STAs that are considered are divided, too. The proposed solution is compared with an optimal solution found by exhaustive search, with a suboptimal “user-pairing resource allocation” algorithm designed for LTE uplink [30], and with a greedy algorithm that splits the channel into equal-size RUs, the number of which equals to the number of users. In the studied scenario, the recursive algorithm outperforms the other considered suboptimal solutions and does not lose to the exhaustive search. Although the authors consider that the same-size RUs can have different conditions, e.g., caused by frequency-selective fading, they consider only downlink transmissions and thus leave the transmission power and MCS control out of consideration, and while solving the scheduling task, assume that for each STA and RU, the STA uses the best MCS allowed by the signal-to-noise ratio. By contrast, we focus on the power control and MCS selection problem while scheduling OFDMA resources. We show that it is essential to consider the dynamics of the channel while scheduling resources.

To sum up, many studies of OFDMA in 802.11ax focus on OFDMA random access and do not consider the problem of scheduled transmissions or consider it in a very simplified form, while the existing research on schedulers for 802.11ax disregards the frequency selective and non-stationary properties of the channel, assumes ideal knowledge of CSI at the AP, and omits the power control aspect of uplink OFDMA transmissions. In the present work, we fill this gap, designing a scheduler that can distinguish between the channel conditions in RUs located in different positions in the frequency band and developing a solution to control the transmission power of STAs to let them compensate possible changes in channel attenuation. In our study, we take into account the fact that the AP does not always have actual information about the CSI and buffer status for all the STAs.

## 4. Problem Statement

**Figure 3:**
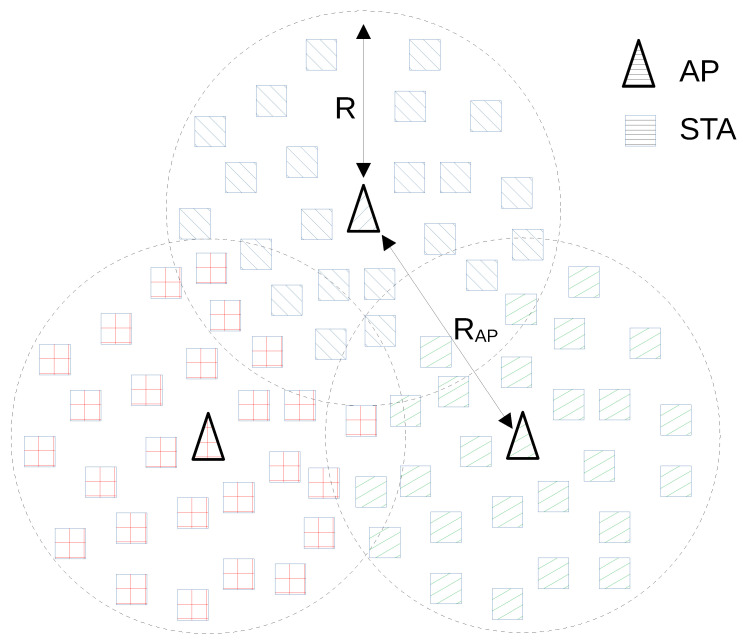
Studied network scheme: different kinds of hatching show different basic service sets.

Consider a network with several APs. Let *R* be the maximum allowable distance between an AP and a STA associated with it. The STAs are uniformly distributed in an area consisting of circles of radius *R* around all APs, as shown in Figure 3. All key notations used in this paper are summarized in Table 2.

Every STA is associated with the closest AP. At random time instances, the STAs generate data flows that should be transmitted to the corresponding APs. After generating a flow, a STA transmits a BSR to inform the AP that this STA has data for the transmission.

The AP allocates resources to the associated STAs that have sent BSRs. Note that every AP makes resource allocation independently of the other APs. Let $\left\{r_{j}\right\}$ be the set of all RUs, which differ from each other in size and location in the band. For the 40 MHz band, all $r_{j}$ are numbered in Figure 1. Based on the RU structure, we determine the inclusion and intersection operations of different RUs.

$$\begin{array}{cc} \; & \mathrm{Inclusion}:\;r_{i}\subset r_{j}\;\mathrm{if}\;r_{j}\;\mathrm{contains}\;\mathrm{all}\;r_{i}\;\mathrm{frequencies},\\ \; & \mathrm{Intersection}:\;r_{i}\cap r_{j}=\left\{\begin{array}{c} r_{i},\;r_{i}\subset r_{j},\\ r_{j},\;r_{j}\subset r_{i},\\ \emptyset ,\;r_{i}\neg \subset r_{j}\;\mathrm{and}\;r_{j}\neg \subset \;r_{i}. \end{array}\right. \end{array}$$

Note that there are no partially overlapping RUs. If two RUs contain the same frequencies, it means that one of them fully contains the other one. For the RU structure from Figure 1: $r_{2}\subset r_{26}$; $r_{26}\subset r_{30}$; $r_{19}\cap r_{26}=r_{19}$.

Let $S={\left\{s_{i}\right\}}_{i=1}^{n}$ be the set of STAs that have data to transmit. The RU allocation to STAs is the set of pairs $X=\{\left(s_{i},r_{j}\right)\}$, where $\left(s_{i},r_{j}\right)$ means that STA $s_{i}$ was assigned to RU $r_{j}$. To simplify the notation, we further write s∈X, meaning that such a pair (s,r) exists, that (s,r)∈X. Similarly, we write r∈X, meaning that such a pair (s,r) exists, that (s,r)∈X.

Using the above notations, we determine the *restrictions* on the RU allocations:

(1) $$\begin{array}{cc} \; & \forall \left(s_{i},r_{i}\right),\left(s_{j},r_{j}\right)\in X,r_{i}\neq r_{j}\to s_{i}\neq s_{j},\\ \; & \forall \left(s_{i},r_{i}\right),\left(s_{j},r_{j}\right)\in X,s_{i}\neq s_{j}\to r_{i}\cap r_{j}=\emptyset , \end{array}$$

where the first restriction means that a STA cannot be allocated two RUs at once, and the second restriction means that the RUs should not intersect with each other.

In addition to RU allocation, the AP assigns MCSs and transmit powers. As noted in Section 2, parts of one OFDMA frame from different STAs have to reach AP with approximately equal power. To satisfy this requirement, we make the STAs involved in the same OFDMA transmission use the same MCS. In this case, power equalizing can be easily done. Suppose that the RU allocation is built, and one of the MCSs was chosen. If the AP knows channel conditions, it can compute the required transmit power and, consequently, the received power for each STA. The AP finds the minimum received power value, and then the STAs decrease their transmit power so that the received power would be equal to this minimum. If the STAs transmit at the different MCSs, a more complex equalizing procedure is required. Note that the STAs can change not only the transmit powers but also the MCSs.

To state the allocation problem, we introduce a utility function *U* that indicates the quality of resource allocation. The scheduler should select the allocation that maximizes this function. Let *C* be the set of available MCSs. Let λ(s,r,c) be the gain of the utility function if the scheduler assigns a STA *s* to an RU *r* at an MCS c∈C (see Section 5.2 for specific examples of utility function). If the STA does not support transmission in the RU *r* at the MCS *c* (for example, due to the lack of power), then λ=0. Let *H* be the set of all RUs to STAs allocations satisfying the conditions (1). Therefore, the problem of maximizing the utility function can be written as

(2) $$\max_{c\in C}\max_{X\in H}\sum_{(s,r)\in X}\lambda \left(s,r,c\right).$$

Please note that RUs within an allocation X∈H can differ in size (i.e., include a different number of tones). All RUs differ from each other due to frequency selective fading. Therefore the solution of (2) requires a full search for all X∈H, which is highly computing-intensive. A common way to solve hard optimization problems is to use a greedy heuristic, e.g., as it is done in [31]. Therefore, in this paper, we propose a greedy procedure for an approximate solution of (2).

## 5. Scheduling

### 5.1. Greedy Algorithm

Let us, first, show a brief outline of the designed algorithm. All the STAs are sorted in the descending order of increments λ(s,r,c) of the utility function. After that, the first STA in the list is assigned to the widest available RU, i.e., such an RU that the STA’s increment λ(s,r,c) is non-zero. Then, the second STA is assigned to the widest of the remaining RUs, etc. This process continues until the list of STAs or the list of RUs are exhausted. The result is a set of assignments at fixed MCS *c*. The AP builds such sets for each MCS from *C* and then selects the best one. Let us consider this algorithm in more detail (see Algorithm 1).

First, let us fix the MCS *c*. Then, the gain λ(s,r,c) of the utility function for $r\in R_{242}$ is calculated for each STA, where $R_{242}$ is the set of 242-tone RUs of the channel (242-tone RU corresponds to 20 MHz band). Channel conditions for these RUs can differ significantly because of the frequency selective fading. We choose the maximum gain as follows:

(3) $$\lambda_{sort}\left(s\right)=\max_{r\in R_{242}}\lambda (s,r,c).$$

The sorting is done according to the 242-tone RUs as a result of trade-off. On the one hand, these RUs are quite large, and therefore, the $\lambda_{sort}$ values characterize the “average” channel quality. On the other hand, we do not consider larger RUs because the average channel properties in a very wide RU poorly represent the potential of OFDMA, but we still want to exploit the OFDMA feature of dividing the channel by frequency.

**Algorithm 1** Greedy Scheduling Algorithm.
1: Parameters:2: $R_{size}$: the set of size-tone RUs in the channel3: ChannelWidth: number of tones in full channel4: Output:5: $X_{best}\leftarrow \emptyset$: resulting STA-RU assignment6: $gain_{best}\leftarrow 0$: cumulative metric gain for the resulting assignment7: $c_{best}$: MCS for the resulting STA-RU assignment8: **for all** c∈C **do**9: 

     X←∅

10:    **for all**  s∈S **do**11:  
$\;\lambda_{sort}\left(s\right)\leftarrow \max_{r\in R_{242}}\lambda (s,r,c)$12:    **end for**13:    Sort STAs in the descending order of $\lambda_{sort}$ in *S*14:    **while** i≠|S| **or** there are unoccupied RUs **do**15:        **for** size← ChannelWidth **to** 26-tones **do**16:             **for all** $r\in R_{size}$ **and** *r* is unoccupied **do**17:                  **if** $\lambda (s_{i},r,c)>0$ **then**18:                   {$\lambda (s_{i},r,c)>0$ if STA $s_{i}$ is able to transmit to the AP in RU *r* at MCS *c*}19:         $\;X\leftarrow X\cup \{s_{i},r\}$20:                   **goto** label21:             **end if**22:           **end for**23:         **end for**24:         label:25:  **end while**26:

$\;gain\leftarrow \sum_{(s,r)\in X}\lambda \left(s,r,c\right)$

27:  **if** $\;gain>gain_{best}$ **then**28:

$\;X_{best}\leftarrow X$

29:

$\;c_{best}\leftarrow c$

30:

$\;gain_{best}\leftarrow gain$

31:   **end if**32:
**end for**



The STAs are sorted in the descending order of $\lambda_{sort}$. The first STA in this list (i.e., the STA with the largest utility function gain) is assigned to the widest RU in which this STA can transmit at MCS *c* (i.e., its increment λ(s,r,c) is non-zero for the considered RU *r* and MCS *c*). The second STA is assigned to the widest remaining available RU, i.e., the second STA has to have enough power to transmit in this RU, and the resulting assignment *X* of two STAs should satisfy restrictions (1). This procedure is repeated for all the STAs in the list until the end of the list, or until there are no available RUs for all the remaining STAs.

For the resulting assignment *X*, we calculate the cumulative gain:

(4) $$\sum_{(s,r)\in X}\lambda \left(s,r,c\right).$$

The algorithm builds allocations for every MCS in the same way. After that, the algorithm chooses such MCS *c* and respective assignment *X* that the value of the total utility function (4) is maximal.

The algorithm contains a cycle through all MCSs, and for each MCS, the algorithm performs a search on all STAs, which is limited either by the number of STAs or by the number of available RUs. Assuming that the calculation of utility function values is done in constant time, the complexity of the algorithm is $O\left(\left|C\right|\times \min\left\{N,M\right\}\right)$, where *M* is the number of RUs in the considered frequency band and |C| is the number of MCSs in the MCS set. Thus, we see that the proposed greedy approach is much faster than an exhaustive search algorithm which can find an optimal solution but requires a cycle through all MCSs, and all possible RU allocations for all STAs, resulting in the complexity of $O\left(\left|C\right|\times M^{N}\right)$.

### 5.2. Priority Calculations

Our greedy algorithm uses λ as priorities: the STAs with higher λ values obtain more resources. Such priorities can be calculated in different ways. In this paper, we consider 802.11ax variants of three very popular schedulers.

The simplest method is called Max Rate (MR), and its goal is to maximize the network throughput. MR aims to transmit the largest amount of data regardless of the specific STAs that will transmit. Utility function increment λ for MR is defined as follows:

(5) λ(s,r,c)=rate(r,c),

where rate(r,c) is the transmission rate in the RU *r* at the MCS *c*, corresponding to Table 1. As mentioned before, λ(s,r,c)=0 if STA *s* cannot transmit in RU *r* at MCS *c*. This case is not mentioned in the expressions for λ to simplify the notation. Hereinafter, we assume that the data flows transmitted by the STAs are long enough so that we can neglect the possible effects caused by the variance of flow durations at the STAs, e.g., when STAs that transmit long flows and very short flows are served simultaneously using OFDMA.

The next approach is called Proportional Fair (PF). This scheduler takes into account not only the amount of data that the STA can transmit, but also the amount of data that the STA has already transmitted by this moment. Therefore, the STA that has less transmitted data gets higher priority, and λ is calculated as

(6) $$\lambda \left(s,r,c\right)=\frac{rate(r,c)}{Q(s)},$$

where Q(s) is the average data rate (service rate).

The last considered scheduler is Shortest Remaining Processing Time (SRPT). It aims to minimize the flow transmission time, i.e., the time from the flow arrival to the moment the last part of the flow is transmitted. In this paper, we consider the following version of SRPT [8]. The STAs are first sorted by the following values:

(7) $$\lambda_{order}\left(s\right)=\frac{D(s)}{rate(r_{entire},c_{best})},$$

where D(s) is the remaining amount of data in the flow, $r_{entire}$ is the RU which occupies the entire channel, and $c_{best}$ is the fastest MCS at which STA can transmit in $r_{entire}$. The sorting value $\lambda_{order}\left(s\right)$ in that case is the time needed to transmit the flow if the STA is allocated the entire channel. Small $\lambda_{order}\left(s\right)$ value means that the STA *s* is capable of transmitting the remaining data quickly, so sorting for a greedy algorithm should be in the ascending order (not the descending one as in previous cases).

The sorting value $\lambda_{order}\left(s\right)$ does not depend on MCS, so the sorting order is the same for all MCSs. In addition, after the RU assignment if constructed, instead of (4) the scheduler considers the following total utility function which should be optimized:

(8) $$\sum_{(s,r)\in X}\frac{D\left(s\right)-\min\{D\left(s\right),\tau_{max}\times rate\left(r,c\right)\}}{rate(r_{entire},c_{best})}+\sum_{(s,r)\notin X}\frac{D(s)}{rate(r_{entire},c_{best})},$$

where $\tau_{max}=5484$μs is the maximal transmission time according to the standard. The product $\tau_{max}\times rate\left(r,c\right)$ is the maximal amount of data which the STA can transmit if it is assigned to RU *r* at MCS *c*. Therefore, the numerator of the left term is the amount of data which remains after transmission, i.e., it is the new value of D(s). Therefore, expression (8) defines the total time to transmit all flows if each STA is assigned to the entire channel RU. The scheduler chooses such an MCS *c* and a respective assignment *X* that the value of (8) is minimal.

## 6. Power Control

According to the standard, for the trigger-based transmissions, the *STA* determines its transmit power in the following way:

(9) $$P_{TX}^{STA}=PL+Target_{RSSI},$$

where PL is the path loss which is measured according to the received TF because the *STA* knows the power with which the AP transmits the TF and assumes that the channel is reciprocal, and $Target_{RSSI}$ is the desired power level of the transmitted signal at the AP. Note that these power calculations are carried out, taking into account the size of the scheduled RUs. $Target_{RSSI}$ is defined by the AP and sent in the TF. The simplest approach is to set high $Target_{RSSI}$ values, to make STAs transmit at the maximum power. However, as noted above, the power level of all received signals in one OFDMA frame shall be approximately the same.

### 6.1. CSI Adaptation Algorithm

Various RUs experience different signal attenuation due to frequency selective fading. This attenuation can be expressed as

(10) $$PL\left(s,r\right)=PL\left(s_{dist}\right)+P\left(r_{size}\right)+Fading\left(s,r\right)+const$$

where $PL(s_{dist})$ is the signal attenuation caused by the distance between the AP and the STA *s*, $P(r_{size})$ shows the required power for a particular RU size (the smaller is the RU, the less power is required), and Fading(s,r) is a fading component. The AP knows PL(s,r) values for each *s* and *r*, so it is able to determine the possibilities of the STA *s* to transmit in RU *r* at MCS *c*. The allocation is constructed based on this information. However, Fading(s,r) values known to the AP may differ from the actual values.

Let F(s,r) be an estimate of Fading(s,r) value at the AP. Let the information about fading on each STA (F(s,r) values) be updated on the AP with a period $T_{fd}$ using channel sounding procedure. Then, some time after the last update, the estimate F(s,r) might be out of date. In this case, the AP could allocate to the *STA* such RU and MCS that the transmission will fail, the data will be lost because of deteriorating channel conditions, and the *STA* will have to retransmit.

To reduce the number of retransmissions, we propose a closed-loop CSI Adaptation Algorithm that improves the estimate F(s,r) based on the results of past transmissions. The algorithm has the following idea. If the STA *s* fails to transmit in the RU *r* for several times in a row, it means that the channel conditions have deteriorated compared to the last update, and therefore the estimate F(s,r) is increased for this *STA* and RU. The reverse is also true: if the *STA* makes several consecutive successful transmissions, it reduces the F(s,r).

Let us describe the CSI Adaptation Algorithm in detail, see Algorithm 2.

We use the following notations. Let $F_{\left\{T_{fd}\right\}}\left(s,r\right)$ be the Fading(s,r) at the time of the last update. For each STA *s* and each RU *r*, we introduce counters of successful and failed transmissions $count_{success}\left(s,r\right)$ and $count_{fail}\left(s,r\right)$. Furthermore, add(s,r) is a variable which indicates how $F_{\left\{T_{fd}\right\}}\left(s,r\right)$ should be changed. According to (10), increase (decrease) of this value means increase (decrease) of pathloss PL(s,r).

Initially, all the mentioned variables are equal to zero.

After F(s,r) values are obtained, the new PL(s,r) values are calculated. Thus, at the next execution of the scheduling algorithm, it uses new PL(s,r) values.

Note that the parameters $e_{mid}$ and $e_{up}$ are needed to provide fast recovery (when $e_{mid}<e_{up}$) of F(s,r) if its value was increased before. The variables $count_{success}\left(s,r\right)$, $count_{fail}\left(s,r\right)$ and add(s,r) are set to zero when actual fading information is received (when $F_{\left\{T_{fd}\right\}}\left(s,r\right)$ is updated).
**Algorithm 2** CSI Adaptation Algorithm.1:Parameters:2:$e_{down}$: number of failed transmissions in a row to increase F(s,r)3:$e_{mid}$ and $e_{up}$: number of successful transmissions in a row to decrease F(s,r)4:step: measure of F(s,r) change5:Let *s* be assigned to *r* by the greedy algorithm6:**if** Transmission failed **then**7:$\;count_{fail}\left(s,r\right)\leftarrow count_{fail}\left(s,r\right)+1$8:$\;count_{success}\left(s,r\right)\leftarrow 0$9:   **if** $\;count_{fail}\left(s,r\right)\geq e_{down}$ **then**10:$\;count_{fail}\left(s,r\right)\leftarrow 0$11:     add(s,r)←add(s,r)+112:   **end if**13: **else**14:$\;count_{success}\left(s,r\right)\leftarrow count_{success}\left(s,r\right)+1$15:$\;count_{fail}\left(s,r\right)\leftarrow 0$16:   **if**  add(s,r)>0 **then**17:$\;e\leftarrow e_{mid}$18:   **else**19:$\;e\leftarrow e_{up}$20:   **end if**21:   **if** $\;count_{success}\left(s,r\right)\geq e$ **then**22:$\;count_{success}\left(s,r\right)\leftarrow 0$23:     add(s,r)←add(s,r)−124:   **end if**25: **end if**26: $F\left(s,r\right)=F_{\left\{T_{fd}\right\}}\left(s,r\right)+add\left(s,r\right)\times step$

### 6.2. Power Supplement

Another possible approach to deal with the sudden worsening of the channel conditions caused by the fading is to maintain some reserve in transmission power. We denote this approach as Power Supplement and implement it as follows. We define a parameter $P_{sup}$. With Power Supplement, when the AP sends a $Target_{RSSI}$ parameter to a STA, it increases this parameter by $P_{sup}$. As a result, according to (9), the STA increases its transmission power by $P_{sup}$ (provided that it does not reach the power limit).

The idea behind this approach is that when the AP schedules transmission for a STA at a given MCS, it expects that the power of the STA signal at the AP will be sufficient for reliable reception of the signal at the considered MCS. With Power Supplement, we increase the received power but do not change the MCS, so that if the fading leads to a sudden drop of the received power, it still will be enough for correct reception.

Note that such an approach can be combined together with the CSI Adaptation Algorithm described earlier.

## 7. Numerical Results

The general model of the system is described in Section 4. We consider a 40 MHz channel in the 5 GHz band. The RU structure is shown in Figure 1. The flow sizes are taken from a truncated lognormal distribution with minimal, average, and maximal values of 100 KB, 3 MB, and 100 MB, respectively. The arrival time of the new flow is taken from the truncated exponential distribution with minimal, average, and maximal values of 1 s, 3 s, and 6 s, respectively. For simplicity, the maximum power difference for all experiments is 10 dB and equal for all MCSs.

To calculate $PL(s_{dist})$ from (10), we use the following signal attenuation model [32]:

(11) $$\begin{array}{cc} PL(s_{dist}) & =40.05+20\log_{10}\left(f_{c}/2.4\right)+20\log_{10}\left(min\left(s_{dist},5\right)\right)+\\ \; & +I\left(s_{dist}>5\right)\times 35\log_{10}\left(s_{dist}/5\right) \end{array}$$

where $f_{c}$ is the center frequency in GHz, I is the indicator function, and $s_{dist}$ is the distance from STA to the AP. This formula shows the dependency of the path loss (expressed in dB) on the distance.

As we consider a 40 MHz channel, we take into account the RU sizes as follows:

(12) $$P\left(r_{size}\right)=10\log_{10}\left(r_{size}/18\right)$$

where $r_{size}$ is the number of 26-tone RUs, from which *r* consists of according to Figure 1. For example, the 52-tone RU consists of two 26-tone RUs, 242-tone RU consists of 9 and the maximal value is 18 (484-tone RU). The Fading(s,r) values are calculated using the TGac D NLOS [33] model recommended for 11ax simulation [32].

In our simulation, we place three APs at the vertices of an equilateral triangle with a side $R_{AP}=50$ m. The maximum distance between STA and AP is R=30 m.

The greedy algorithm has been implemented for the experiments for the MR, PF, and SRPT utility functions described in Section 5. We also consider schedulers from [8] that do not divide the channel by frequency for the comparison.

In the first series of experiments, we examine the performance of

- the proposed schedulers (denoted as greedy),
- the schedulers that perform an exhaustive search over all STA-RU allocations and thus obtain an optimal solution of the optimization task (2) (denoted as exhaustive), and
- the schedulers that do not divide the channel by frequency (denoted as legacy).

**Figure 4:**
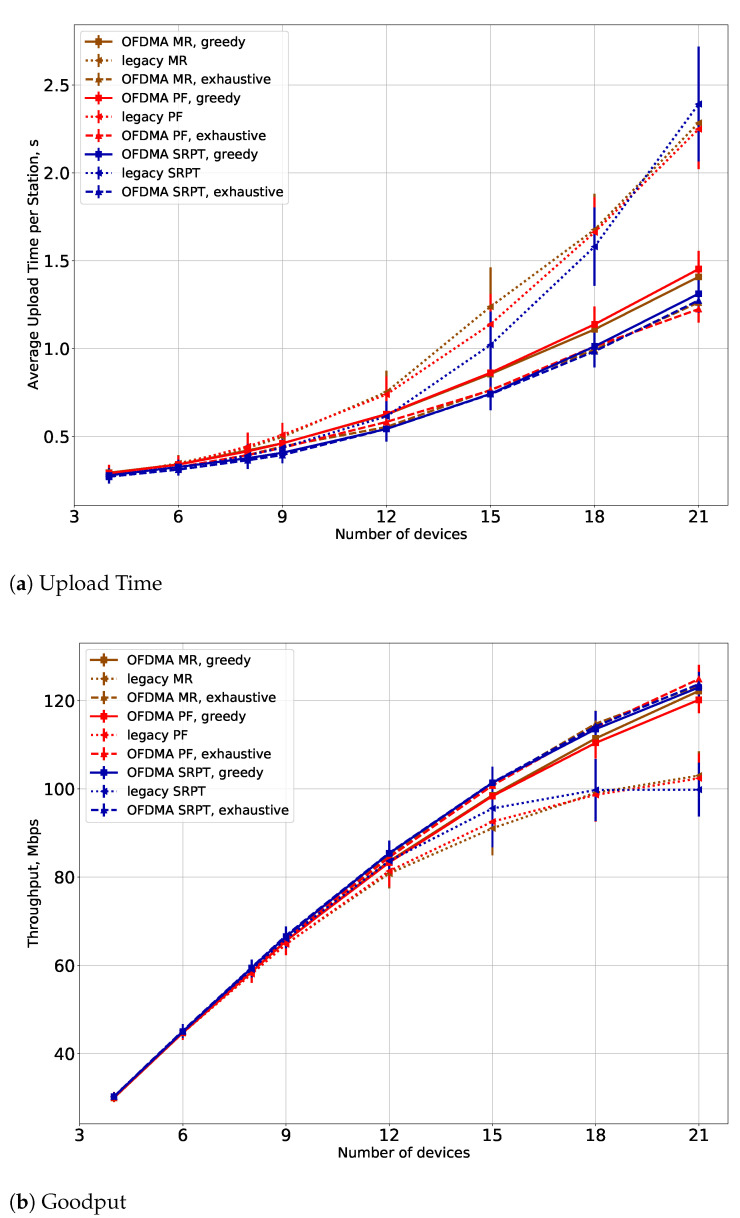
(**a**) Upload time and (**b**) goodput for the proposed greedy schedulers in comparison with optimal solution and legacy schedulers for small numbers of STAs.

The results are shown in Figure 4 in case $T_{fd}=0\;s$, i.e., when the APs always know the relevant channel conditions. The average upload time is the average time to transmit a flow, and goodput is the overall throughput of the system. The total number of STAs equals *N*. Note that all APs work simultaneously, so there may be several parallel OFDMA transmissions (in the case when TFs are transmitted at the same time or when an AP cannot receive TF from the other AP successfully). The results show that the developed OFDMA schedulers significantly outperform the legacy ones: the average upload time is almost two times lower, and the throughput is approximately 20% higher. At the same time, the proposed greedy scheduler, although it is a heuristic approach, does not degrade performance much with respect to the optimal solution. The loss in the average upload time is approximately 10%, and the loss in throughput is approximately 5%, but the computation time is much lower.

**Figure 5:**
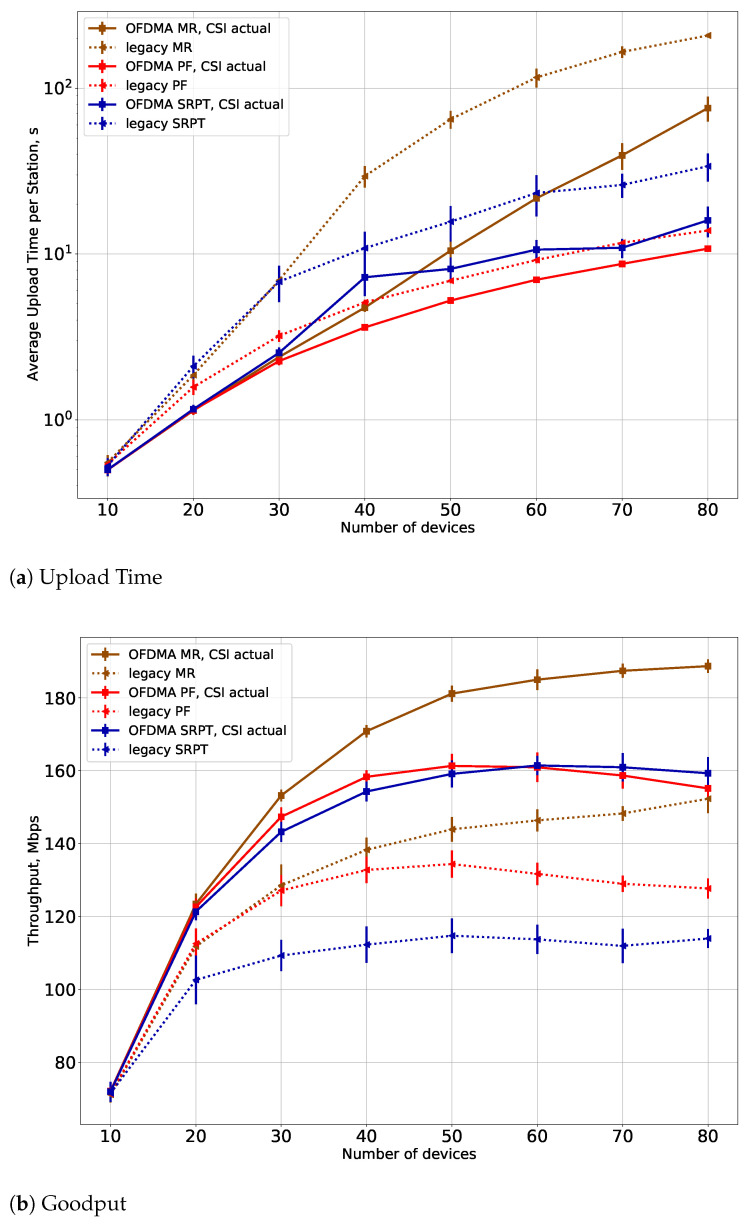
(**a**) Upload time and (**b**) goodput for the proposed schedulers in comparison with the legacy schedulers for high numbers of STAs.

In Figure 4, we show results only for small numbers of STAs, up to N=21, because it takes too much time to obtain results for the exhaustive search schedulers. In Figure 5, we show results for bigger numbers of STAs for the greedy schedulers and the legacy schedulers. The results show that even for big numbers of STAs, the proposed greedy schedulers have a significant gain in goodput and the average upload time. An important result is that the gain in average upload time is more significant for small numbers of STAs, and the gain in throughput becomes more significant for bigger numbers of STAs. This result corresponds to one reported in [28] and is explained by the fact that the network becomes saturated in the case of big numbers of STAs.

**Figure 6:**
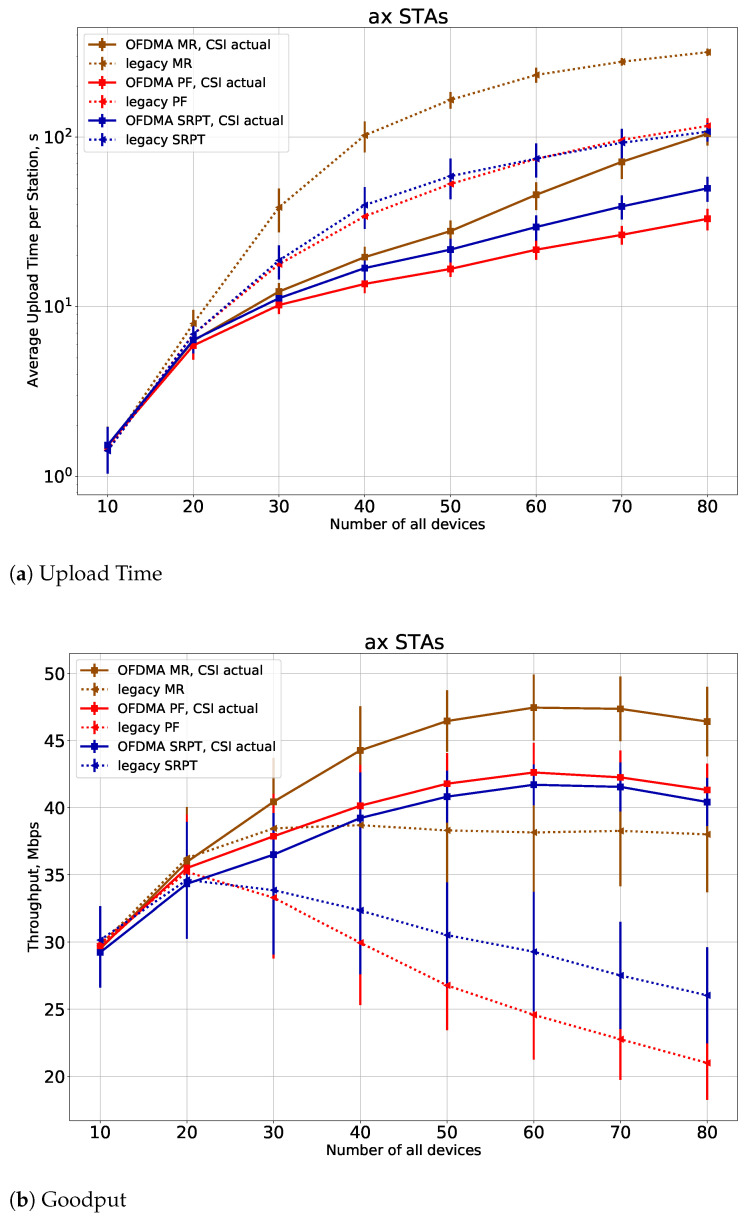
(**a**) Upload time and (**b**) goodput of the *ax* STAs if the half of all devices are *legacy*.

In the second series of experiments, we investigate the impact of *legacy* STAs on the system performance. The difference between legacy STAs and the previously considered STAs (*ax* STAs) is that *legacy* STAs do not use scheduled access via trigger frames. These STAs only use conventional random access with default parameters and transmit data instead of transmitting BSRs and waiting for TFs. The *legacy* STAs use the whole channel and the maximum available power for the transmission. In Figure 6, we show the performance of the *ax* STAs in the system where half of all STAs are *legacy*. The results show that the presence of *legacy* STAs degrades the performance of *11ax* STAs, which can be solved by a proper configuration of the channel access parameters, as done in [34]. However, the relative gain of the proposed greedy schedulers against legacy schedulers remains of almost the same order as without *legacy* STAs.

**Figure 7:**
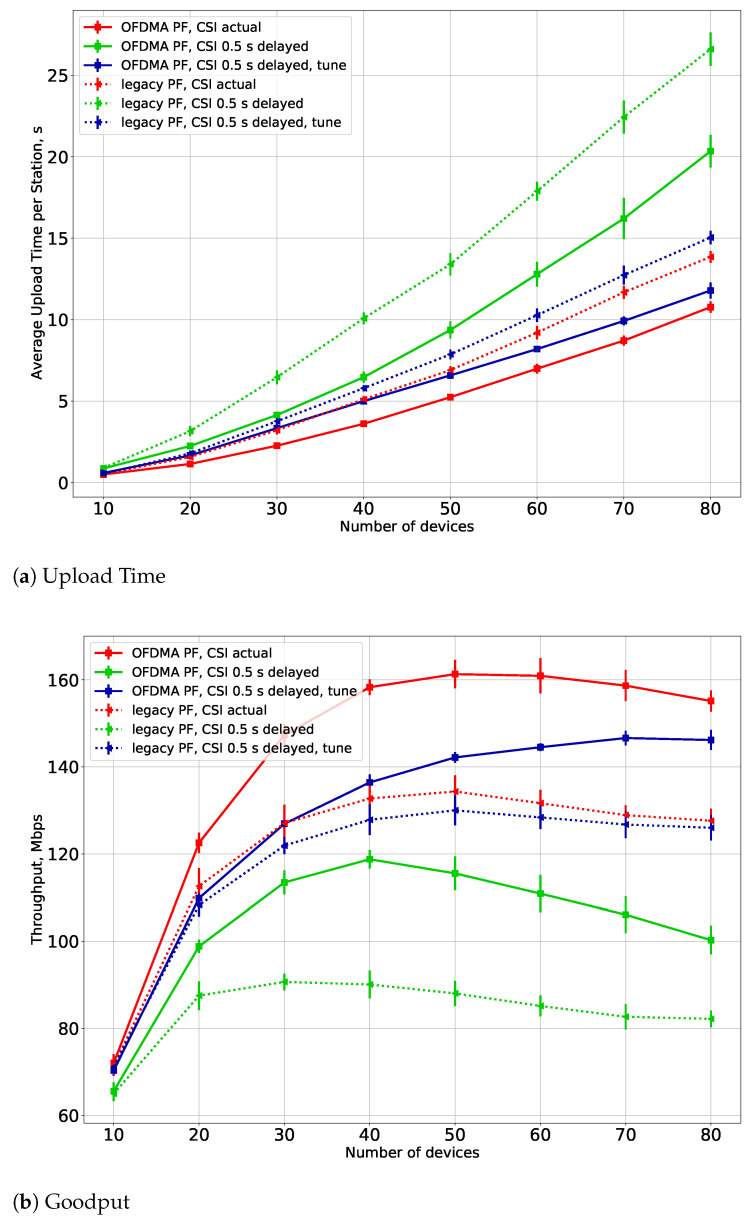
(**a**) Upload time and (**b**) goodput for the CSI Adaptation Algorithm for FS-PF.

In the third series of experiments, we check the efficiency of the CSI Adaptation Algorithm (see Section 6). The results are shown in Figure 7 for different variants of the greedy-PF scheduler. We consider the cases with $T_{fd}=0\;s$, i.e., when the APs always have actual information about the channel, and the $T_{fd}=0.5\;s$, i.e., when fading information is updated every 0.5 s. Lines “tune” and “no tune” indicate whether the CSI Adaptation Algorithm is used or not, respectively. From the results, we can see that the adaptation gives a significant performance advantage both in terms of the average upload time and of the goodput. Moreover, the average upload time in using the adaptation is pretty close to the case when the APs know the actual channel information.

**Figure 8:**
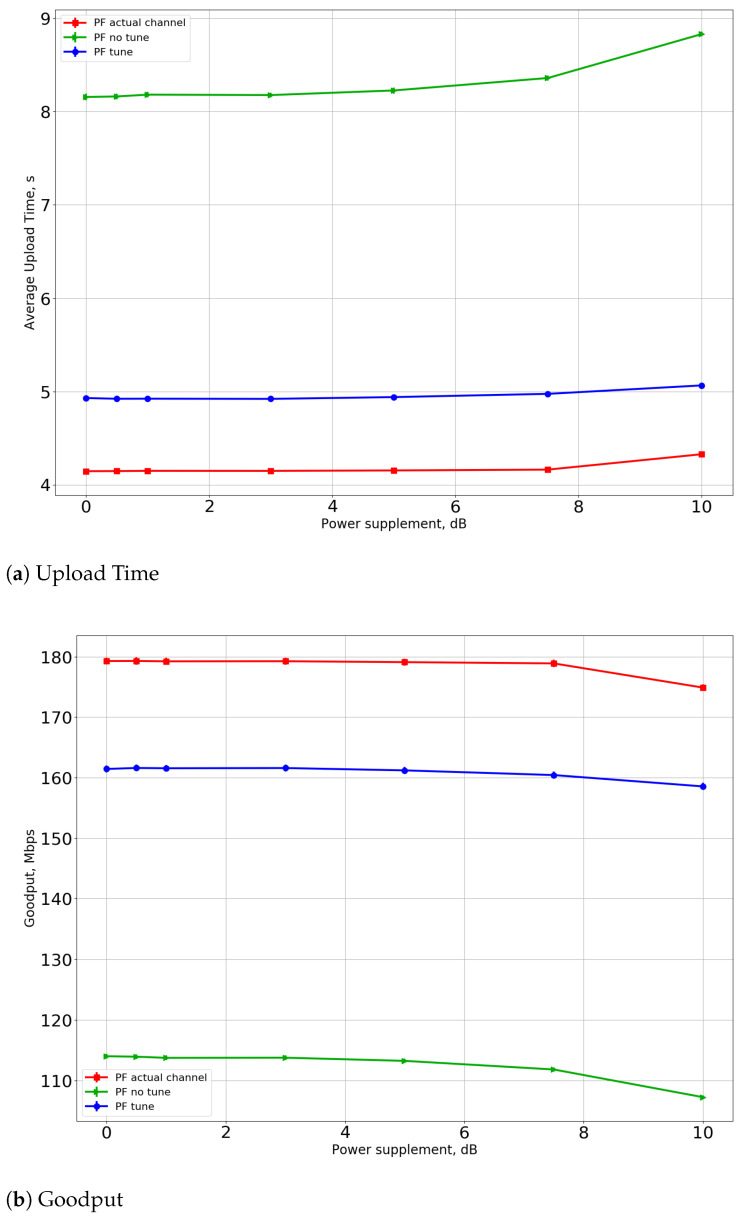
(**a**) Upload time and (**b**) goodput for power supplement for R=20 m.

**Figure 9:**
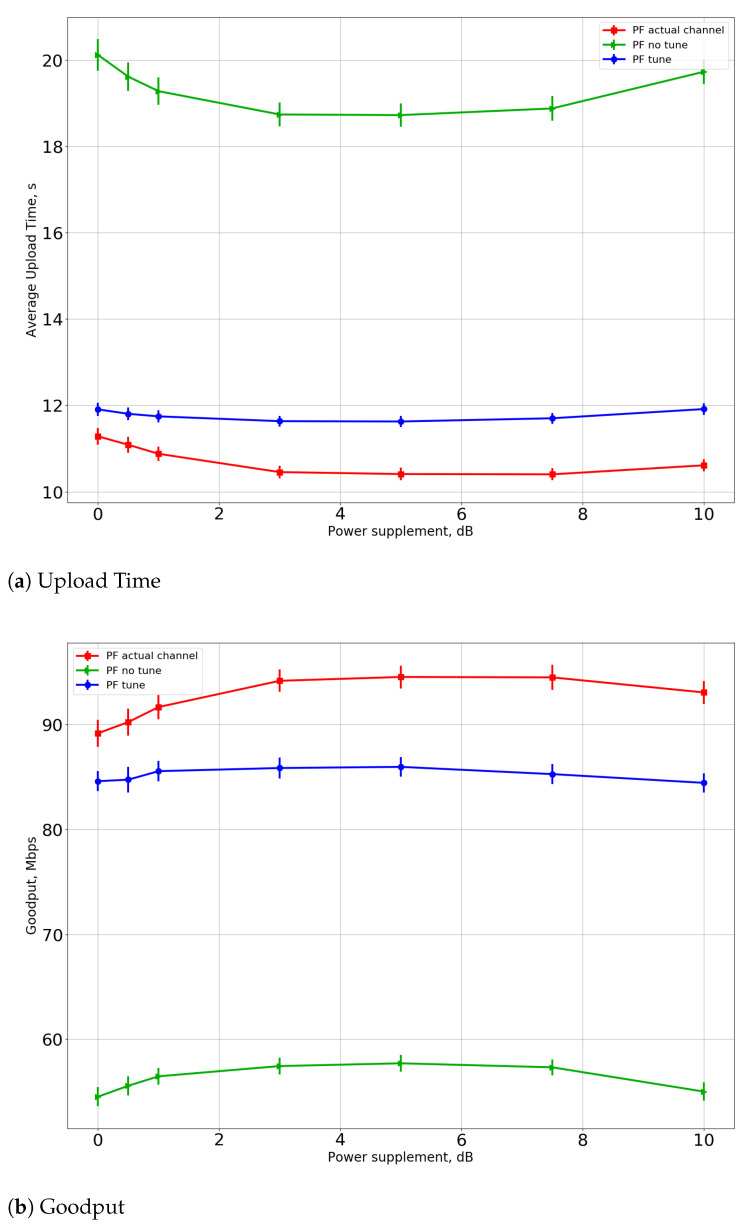
(**a**) Upload time and (**b**) goodput for power supplement for R=40 m.

We also investigate the effect of the power supplement described in Section 6.2. In this series of experiments, we also place three APs with $R_{AP}=20\;m$ and fix the total number of STAs N=50. The dependencies of the upload time and the goodput on the $P_{sup}$ parameter value for the greedy-PF scheduler for R=20 m and R=40 m (MR and SRPT have similar results) are shown in Figure 8 and Figure 9. For the R=20 m case, power supplement only worsens the performance because of the maximum power difference constraint and because the STAs are located not so far from each other. However, for the R=40 m case, increasing the transmit power has a beneficial effect on performance. Such an effect happens due to the interference at the AP from far devices that are not regulated by traditional random access. Furthermore, we see that performance improvement for the “tune” case is relatively small. It means that the number of transmission errors caused by interference also is reduced by the CSI Adaptation Algorithm.

## 8. Conclusions

In this paper, we have studied the uplink OFDMA transmissions under conditions of frequency-selective fading. We have proposed a greedy approach of RU assignment for three different metrics: MR, PF, and SRPT. It has been shown that this approach works better in terms of throughput and average flow upload time than algorithms that do not divide the channel by frequency. We also have shown that the proposed approach retains its advantage if there are a lot of conventional devices in the system.

We have introduced an algorithm to adaptively change the information about channel conditions when this information is updated with a certain period. We have shown that this algorithm also increases the throughput and reduces the upload time because the AP has more relevant data on the quality of the channel for STAs when scheduling channel resources.

We have also shown that an unconditional increase of transmit power to counter the possible effects of fading is almost useless when the proposed adaptation algorithm is used and can improve or worsen the performance depending on the scenario.

As a direction of future work, we consider extending the proposed solutions to uplink OFDMA with MU-MIMO. Such an extension is complicated by many issues, including timely sounding, CSI estimation [27,35] and overhead reduction. Furthermore, we plan to extend our results to a bit more flexible RU structure of 802.11be [36], which is currently being developed. 

## Figures and Tables

**Table 1:** Data rates in RUs with different number of tones, Mbps. Reprinted with permission from [8]. Copyright 2018 IEEE.

#	MCS	26-Tone	52-Tone	106-Tone	242-Tone	484-Tone	996-Tone
1	BPSK, 1/2	0.8	1.7	3.5	8.1	16.3	34
2	QPSK, 1/2	1.7	3.3	7.1	16.3	32.5	68.1
3	QPSK, 3/4	2.5	5	10.6	24.4	48.8	102.1
4	16-QAM, 1/2	3.3	6.7	14.2	32.5	65	136.1
5	16-QAM, 3/4	5	10	21.3	48.8	97.5	204.2
6	64-QAM, 2/3	6.7	13.3	28.3	65	130	272.2
7	64-QAM, 3/4	7.5	15	31.9	73.1	146.3	306.3
8	64-QAM, 5/6	8.3	16.7	35.4	81.3	162.5	340.3
9	256-QAM, 3/4	10	20	42.5	97.5	195	408.3
10	256-QAM, 5/6	11.1	22.2	47.2	108.3	216.7	453.7
11	1024-QAM, 3/4	—	—	—	121.9	243.8	510.4
12	1024-QAM, 5/6	—	—	—	135.4	270.8	576.1

**Table 2:** Notations.

Symbol	Description
add(s,r)	Increment of the fading estimation for STA *s* in RU *r*
*C*	Set of MCSs
$c_{best}$	The fastest MCS at which the STA can transmit in entire channel
$count_{success}\left(s,r\right)$	Number of consecutive successful transmissions from STA *s* in RU *r*
$count_{fail}\left(s,r\right)$	Number of consecutive failed transmissions from STA *s* in RU *r*
D(s)	Remaining amount of data at STA *s*
Fading(s,r)	Fading of the signal from STA *s* in RU *r*
F(s,r)	Estimated values of fading component from STA *s* in RU *r*
$f_{c}$	Carrier frequency
λ(s,r,c)	The gain of the utility function if the scheduler assigns a STA *s* to an RU *r* at an MCS *c*
*M*	Number of RUs in the frequency band
*N*	Number of STAs
*H*	Set of all RU allocations that satisfy the restriction (1)
$P_{TX}^{STA}$	STA transmission power
$P(r_{size})$	Required power for successful reception in RU $r_{size}$
$P_{sup}$	Power supplement: the value by which the STA should increase its transmission power
PL	Path-loss
Q(s)	Average service rate of the STA *s*
*R*	Maximal allowed distance between an AP and an associated STA
$R_{242}$	Set of 242-tone (i.e., 20 MHz-wide) RUs of the channel
$r_{j}$	Resource Unit *j*
$r_{entire}$	RU which occupies the entire channel
rate(r,c)	Transmission rate in RU *r* at MCS *c*
$s_{i}$	STA *i*
$s_{dist}$	Distance from the STA to the AP
*S*	Set of STAs that have data for transmission
$T_{fd}$	Period of fading estimation update
$Target_{RSSI}$	desirable power level of the transmitted signal at the AP
$\tau_{max}$	5484 μs, maximal transmission time
*X*	Set of pairs $\left(s_{i},r_{j}\right)$ which indicates the resource allocation

## Data Availability

Not applicable.

## Abbreviations

The following abbreviations are used in this manuscript:

ACK	Acknowledgment
AP	Access Point
BlockACK	Block Acknowledgment
BPSK	Binary Phase Shift Keying
BSR	Buffer Status Report
CSI	Channel State Information
LAN	Local Area Network
LTE	Long-Term Evolution
MAN	Metropolitan Area Network
MCS	Modulation and Coding Scheme
MIMO	Multiple-Input, Multiple-Output
MR	Max Rate (scheduler)
MU-MIMO	Multi-User Multiple-Input, Multiple- Output
OFDMA	Orthogonal Frequency Division Multiple Access
PF	Proportional Fair (scheduler)
QAM	Quadrature Amplitude Modulation
QPSK	Quadrature Phase Shift Keying
RSSI	Received Signal Strength Indicator
RU	Resource Unit
SIFS	Short Inter-Frame Space
SNR	Signal-to-Noise Ratio
SRPT	Shortest Remaining Processing Time (scheduler)
STA	Station
TCP	Transmission Control Protocol
TF	Trigger Frame
UDP	User Datagram Protocol
UORA	Uplink OFDMA Random Access
WLAN	Wireless Local Area Network

## References

[1] Cisco Annual Internet Report (2018–2023) White Paper. https://www.cisco.com/c/en/us/solutions/collateral/executive-perspectives/annual-internet-report/white-paper-c11-741490.html.

[2] Khorov E., Kiryanov A., Lyakhov A., Bianchi G. (2018). A tutorial on IEEE 802.11ax High Efficiency WLANs. IEEE Commun. Surv. Tutor..

[3] Bellalta B. (2016). IEEE 802.11ax: High-efficiency WLANs. IEEE Wirel. Commun..

[4] IEEE P802.11ax™ Standard for Information Technology—Telecommunications and Information Exchange between Systems—Local and Metropolitan Area Networks—Specific Requirements Part 11: Wireless LAN Medium Access Control (MAC) and Physical Layer (PHY) Specifications Amendment 1: Enhancements for High-Efficiency WLAN. https://standards.ieee.org/standard/802_11ax-2021.html.

[5] Seong K., Mohseni M., Cioffi J.M. Optimal resource allocation for OFDMA downlink systems. Proceedings of the 2006 IEEE International Symposium on Information Theory.

[6] Yaacoub E., Dawy Z. (2011). A survey on uplink resource allocation in OFDMA wireless networks. IEEE Commun. Surv. Tutor..

[7] Lee S.B., Pefkianakis I., Meyerson A., Xu S., Lu S. Proportional Fair Frequency-Domain Packet Scheduling for 3GPP LTE Uplink. Proceedings of the INFOCOM 2009, Rio de Janeiro.

[8] Bankov D., Didenko A., Khorov E., Lyakhov A. OFDMA uplink scheduling in IEEE 802.11ax networks. Proceedings of the 2018 International Conference on Communications (ICC).

[9] Power Control for Multi-User Transmission in 802.11ax. https://mentor.ieee.org/802.11/dcn/16/11-16-0331-01-00ax-power-control-for-multi-user-transmission-in-802-11ax.pptx.

[10] Lanante L., Ochi H., Uwai T., Nagao Y., Kurosaki M., Ghosh C. Performance analysis of the 802.11ax UL OFDMA random access protocol in dense networks. Proceedings of the 2017 IEEE International Conference on Communications (ICC).

[11] Naik G., Bhattarai S., Park J.M. Performance analysis of uplink multi-user OFDMA in IEEE 802.11ax. Proceedings of the 2018 IEEE International Conference on Communications (ICC).

[12] Bai J., Fang H., Suh J., Aboul-Magd O., Au E., Wang X. Adaptive uplink OFDMA random access grouping scheme for ultra-dense networks in IEEE 802.11ax. Proceedings of the 2018 IEEE/CIC International Conference on Communications in China (ICCC).

[13] Lee K.H. (2019). Performance analysis of the IEEE 802.11ax MAC protocol for heterogeneous Wi-Fi networks in non-saturated conditions. Sensors.

[14] Bhattarai S., Naik G., Park J.M.J. Uplink resource allocation in IEEE 802.11ax. Proceedings of the ICC 2019-2019 IEEE International Conference on Communications (ICC).

[15] Daldoul Y., Meddour D.E., Ksentini A. (2020). Performance evaluation of OFDMA and MU-MIMO in 802.11ax networks. Comput. Netw..

[16] Xie D., Zhang J., Tang A., Wang X. (2020). Multi-Dimensional Busy-Tone Arbitration for OFDMA Random Access in IEEE 802.11ax. IEEE Trans. Wirel. Commun..

[17] Lanante L., Ghosh C., Roy S. (2020). Hybrid OFDMA random access with resource unit sensing for next-gen 802.11ax WLANs. IEEE Trans. Mobile Comput..

[18] Avdotin E., Bankov D., Khorov E., Lyakhov A. OFDMA resource allocation for real-time applications in IEEE 802.11ax networks. Proceedings of the 2019 IEEE International Black Sea Conference on Communications and Networking (BlackSeaCom).

[19] Avdotin E., Bankov D., Khorov E., Lyakhov A. Enabling massive real-time applications in IEEE 802.11be networks. Proceedings of the 2019 IEEE 30th Annual International Symposium on Personal, Indoor and Mobile Radio Communications (PIMRC).

[20] Sharon O., Alpert Y. (2017). Scheduling strategies and throughput optimization for the uplink for IEEE 802.11ax and IEEE 802.11ac based networks. Wirel. Sens. Netw..

[21] Taneja M., Sahu B., Murthy R., Ramachandran B., Sharma A., Howlader P. (2018). Resource Allocation in 802.11ax Networks. Tech. Discl. Commons.

[22] Bellalta B., Kosek-Szott K. (2019). AP-initiated multi-user transmissions in IEEE 802.11ax WLANs. Ad Hoc Netw..

[23] Dutta A., Gupta N., Das S., Maity M. MMRU-ALLOC: An optimal resource allocation framework for OFDMA in IEEE 802.11ax. Proceedings of the 2020 IEEE 31st Annual International Symposium on Personal, Indoor and Mobile Radio Communications (PIMRC).

[24] Filoso D.G., Kubo R., Hara K., Tamaki S., Minami K., Tsuji K. Proportional-based resource allocation control with QoS adaptation for IEEE 802.11ax. Proceedings of the ICC 2020-2020 IEEE International Conference on Communications (ICC).

[25] Kuran M.Ş., Dilmac A., Topal Ö., Yamansavascilar B., Avallone S., Tugcu T. Throughput-maximizing OFDMA Scheduler for IEEE 802.11ax Networks. Proceedings of the 2020 IEEE 31st Annual International Symposium on Personal, Indoor and Mobile Radio Communications.

[26] The ns-3 Network Simulator. http://www.nsnam.org/.

[27] Lee K.H. (2019). Using OFDMA for MU-MIMO user selection in 802.11 ax-based Wi-Fi networks. IEEE Access.

[28] Avallone S., Imputato P., Redieteab G., Ghosh C., Roy S. (2021). Will OFDMA Improve the Performance of 802.11 WiFi Networks?. IEEE Wireless Commun..

[29] Wang K., Psounis K. (2020). Efficient scheduling and resource allocation in 802.11ax multi-user transmissions. Comput. Commun..

[30] Fan J., Li G.Y., Yin Q., Peng B., Zhu X. (2012). Joint user pairing and resource allocation for LTE uplink transmission. IEEE Trans. Wirel. Commun..

[31] Saraereh O.A., Alsaraira A., Khan I., Uthansakul P. (2019). An efficient resource allocation algorithm for OFDM-based NOMA in 5G systems. Electronics.

[32] TGax Simulation Scenarios. https://mentor.ieee.org/802.11/dcn/14/11-14-0980-14-00ax-simulationscenarios.docx.

[33] TGac Channel Model Addendum. https://mentor.ieee.org/802.11/dcn/09/11-09-0308-03-00ac-tgac-channel-model-addendum-document.doc.

[34] Khorov E., Loginov V., Lyakhov A. Several EDCA parameter sets for improving channel access in IEEE 802.11ax networks. Proceedings of the 2016 International Symposium on Wireless Communication Systems (ISWCS).

[35] Naser M.A., Alsabah M., Mahmmod B.M., Noordin N.K., Abdulhussain S.H., Baker T. (2020). Downlink Training Design for FDD Massive MIMO Systems in the Presence of Colored Noise. Electronics.

[36] Khorov E., Levitsky I., Akyildiz I.F. (2020). Current status and directions of IEEE 802.11be, the future Wi-Fi 7. IEEE Access.

